# Concurrent chemoradiotherapy degrades the quality of life of patients with stage II nasopharyngeal carcinoma as compared to radiotherapy

**DOI:** 10.18632/oncotarget.14932

**Published:** 2017-02-01

**Authors:** Xin-Bin Pan, Shi-Ting Huang, Kai-Hua Chen, Yan-Ming Jiang, Jia-Lin Ma, Song Qu, Ling Li, Long Chen, Xiao-Dong Zhu

**Affiliations:** ^1^ Department of Radiation Oncology, Cancer Hospital of Guangxi Medical University, Nanning, Guangxi 530021, P.R. China

**Keywords:** nasopharyngeal carcinoma, quality of life, radiotherapy, concurrent chemoradiotherapy

## Abstract

The purpose of this study was to compare the quality of life (QoL) of stage II nasopharyngeal carcinoma (NPC) patients treated with radiotherapy (RT) versus concurrent chemoradiotherapy (CCRT). In a cross-sectional study, these patients were treated with RT (n = 55) or CCRT (n = 51) between June 2008 and June 2013. For all subjects, disease-free survival was more than 3 years. QoL was assessed using the European Organization for Research and Treatment of Cancer Quality of Life Questionnaire-Core 30 (EORTC QLQ-C30) questions and the Head and Neck 35 (EORTC QLQ-H&N35) questions. RT had better outcomes than CCRT for global QoL, functional scales, symptom scales of fatigue and insomnia, financial problems, and weight gain. Survivors receiving 1 cycle of concurrent chemotherapy had worse QoL outcomes than survivors receiving 2 cycles of concurrent chemotherapy. Patients receiving 3 cycles of concurrent chemotherapy had the best QoL outcomes. Thus, CCRT adversely affects the QoL of patients with stage II NPC as compared to radiotherapy.

## INTRODUCTION

Nasopharyngeal carcinoma (NPC) is an endemic disease in southern China. The incidence of stage II NPC has greatly increased with improvements in diagnosis. Radiotherapy (RT) or concurrent chemoradiotherapy (CCRT) are the primary treatment modalities for stage II NPC. CCRT is recommended by the National Comprehensive Cancer Network, but the evidence is weak [1–4]. However, RT is recommended by the Chinese Anti-Cancer Association because CCRT does not improve survival, but increases toxic reactions [5–9]. The best treatment modality is still controversial.

After treatment, the 5-year disease-specific survival rate is as high as 97.3% for stage II NPC [7]. The high survival rate makes QoL increasingly important. Clinicians should pay more attention to QoL because long-term survivors may have problems with swallowing, hearing, and speech, as well as psychological and functional problems. However, previous studies mainly focused on endpoints of overall survival, disease-free survival, or local control rate [1–9]. These endpoints lack information on patients’ experience with treatment-related toxicities or QoL.

We conducted a cross-sectional study to compare the QoL of patients with stage II NPC treated with RT versus CCRT. The result of this study might help clinicians make treatment decisions and provide information to health workers on which health services are most beneficial.

## RESULTS

### Patients

From June 2008 to June 2013, 235 patients with stage II NPC received radical treatment in the Cancer Hospital of Guangxi Medical University. This study excluded 129 patients. Among the excluded patients, 8 were lost to follow-up, 4 received induced chemotherapy, 40 received adjuvant chemotherapy, 5 died, 9 were loco-regional failures, 7 were distant failures, 51 were non-compliant, and 5 did not complete the questionnaire. We included 106 patients treated with RT (n = 55) or CCRT (n = 51). Disease-free survival of all subjects was more than 3 years. Table 1 summarizes patient characteristics.

**Table 1:** Patient characteristics

	RT (n = 55)	CCRT (n = 51)	*P*
Gender			
Male	38 (69.10%)	32 (62.75%)	0.473
Female	17 (30.90%)	19 (37.25%)	0.739
Age (years)			
Median	43	42	0.915
Range	27-68	22-64	
Follow-up (months)			
Median	62	48	0.000
Range	42-89	38-62	
AJCC stage			
T1N1M0	10 (18.18%)	11 (21.57%)	0.827
T2N0M0	19 (34.55%)	5 (9.80%)	0.004
T2N1M0	26 (47.27%)	35 (68.63)	0.249
Chemotherapy			
1 cycle	/	6 (11.76%)	
2 cycles	/	18 (35.29%)	
3 cycles	/	27 (52.95%)	
Radiotherapy			
2D-CRT	33 (60.00%)	14 (27.45%)	0.006
IMRT	22 (40.00%)	37 (72.55%)	0.051

RT: radiotherapy.

CCRT: concurrent chemoradiotherapy.

2D-CRT: two-dimensional conventional radiotherapy.

IMRT: intensity-modulated radiotherapy.

### QoL of RT versus CCRT for the whole group

RT had higher mean scores in global QoL, physical functioning, role functioning, and emotional functioning but lower mean scores in fatigue, insomnia, financial problems and weight gain compared with CCRT (Table 2). Clinically relevant QoL was significant on the scales of role functioning, emotional functioning, fatigue, insomnia, financial problems, and weight gain based on clinical interpretation (difference in mean scores ≥10 points). The result indicates that CCRT adversely affects the QoL of patients with stage II NPC versus RT.

**Table 2:** Mean quality of life scores of RT versus CCRT for the whole group

Scales	RT (n = 55)		CCRT (n = 51)		T-test	*P*
	Mean	SD	Mean	SD		
EORTC QLQ-C30						
Global quality of life	76.67	16.15	67.81	16.92	9.082	0.000
Physical functioning	87.39	17.67	80.26	17.23	2.102	0.038
Role functioning	87.88	18.27	76.80	19.46	3.024	0.003
Emotional functioning	82.73	22.79	71.90	24.55	2.356	0.020
Cognitive functioning	77.88	27.79	69.28	22.20	1.751	0.083
Social functioning	78.79	24.52	73.20	22.63	1.216	0.227
Fatigue	18.59	19.13	28.76	23.85	−2.431	0.017
Nausea/emesis	3.03	7.92	2.29	6.68	0.520	0.604
Pain	10.30	15.21	15.36	14.85	−1.730	0.087
Dyspnea	6.06	12.97	9.15	18.95	−0.986	0.327
Insomnia	21.82	22.42	34.64	28.25	−2.597	0.011
Appetite loss	8.48	16.00	7.19	15.37	0.424	0.672
Constipation	4.85	16.25	4.58	17.66	0.083	0.934
Diarrhea	4.85	14.93	5.88	12.83	−0.381	0.704
Financial problems	27.27	28.03	41.18	27.15	−2.590	0.011
EORTC QLQ-H&N35						
Pain	7.12	12.05	8.17	7.54	−0.532	0.596
Swallowing	14.09	17.41	17.48	15.21	−1.065	0.289
Senses	16.67	16.97	17.32	17.31	−0.196	0.845
Speech	6.26	10.20	5.88	9.78	0.196	0.845
Social contact	14.70	21.03	19.61	19.42	−1.246	0.216
Social eating	7.39	10.79	6.67	10.41	0.353	0.725
Sexuality	33.03	31.99	43.46	29.83	−1.733	0.086
Teeth	27.88	31.27	32.03	25.79	−0.747	0.457
Opening mouth	16.97	23.89	20.26	22.19	−0.733	0.465
Dry mouth	39.39	28.75	39.22	28.83	0.032	0.975
Sticky saliva	4.85	13.48	7.84	19.54	−0.924	0.358
Coughing	10.30	18.00	13.07	16.44	−0.825	0.411
Feeling ill	13.33	19.88	15.69	20.39	−0.601	0.549
Pain killers	5.45	22.92	9.80	30.03	−0.842	0.402
Nutritional supplements	45.45	57.15	58.82	49.71	−1.281	0.203
Feeding tube	0.00	0.00	0.00	0.00	0.000	1.000
Weight loss	5.45	22.92	13.73	34.75	−1.435	0.155
Weight gain	1.82	13.48	35.29	48.26	−4.783	0.000

RT: radiotherapy.

CCRT: concurrent chemoradiotherapy.

SD: standard deviation.

EORTC QOL-C30: European Organization for Research and Treatment of Cancer

Quality of Life Questionnaire-Core 30.

EORTC QOL-H&N35: The EOTRC Quality of Life Questionnaire-Head and Neck 35.

### QoL of RT versus CCRT by different radiotherapy techniques

In the two-dimensional conventional radiotherapy (2D-CRT) subgroup, RT (n = 33) had better QoL than CCRT (n = 14). Differences between the two groups were clinically relevant (Table 3). Moreover, the intensity modulated radiotherapy (IMRT) subgroup had similar results between RT (n = 22) and CCRT (n = 37) (Table 4). Despite the radiation technique used (2D-CRT or IMRT), RT resulted in better QoL versus CCRT.

**Table 3:** Mean values for all scales of RT versus CCRT with 2D-CRT technique

Scales	RT (n = 33)		CCRT (n = 14)		T-test	*P*
	Mean	SD	Mean	SD		
EORTC QLQ-C30						
Global quality of life	69.95	15.30	53.57	11.65	−3.580	0.001
Physical functioning	80.61	19.10	64.29	14.93	−2.843	0.007
Role functioning	80.81	20.46	59.52	19.30	−3.314	0.002
Emotional functioning	74.49	23.75	50.60	26.65	−3.043	0.004
Cognitive functioning	66.67	30.33	47.62	22.51	−2.110	0.040
Social functioning	66.67	24.65	48.81	19.02	−2.417	0.020
Fatigue	26.94	19.84	51.59	17.76	4.012	0.000
Nausea/emesis	4.04	9.35	4.76	7.81	0.253	0.801
Pain	13.64	17.90	28.57	10.19	2.916	0.006
Dyspnea	9.09	15.08	16.67	17.30	1.508	0.139
Insomnia	28.28	20.62	61.90	22.10	5.006	0.000
Appetite loss	14.14	18.69	19.05	17.12	0.843	0.404
Constipation	8.08	20.46	4.76	12.10	−0.564	0.575
Diarrhea	7.07	18.18	14.29	17.12	1.265	0.212
Financial problems	39.39	28.20	57.14	20.37	2.125	0.039
EORTC QLQ-H&N35						
Pain	10.86	14.05	13.10	7.10	0.563	0.576
Swallowing	22.22	18.00	33.93	14.05	2.165	0.036
Senses	23.74	17.69	28.57	17.82	0.855	0.397
Speech	9.43	11.82	13.49	10.83	1.104	0.276
Social contact	24.24	22.57	39.88	18.83	2.275	0.028
Social eating	12.12	11.72	14.29	13.30	0.556	0.581
Sexuality	47.47	30.08	72.62	30.39	2.613	0.012
Teeth	40.40	32.01	54.76	21.11	1.537	0.131
Opening mouth	27.27	25.62	33.33	18.49	0.799	0.429
Dry mouth	54.55	23.30	66.67	18.49	1.896	0.067
Sticky saliva	7.07	16.15	16.67	21.68	1.490	0.152
Coughing	10.10	17.65	21.43	16.57	2.048	0.046
Feeling ill	18.18	20.57	33.33	18.49	2.483	0.019
Pain killers	3.03	9.73	2.38	8.91	−0.214	0.831
Nutritional supplements	21.21	20.10	28.57	12.10	1.544	0.131
Feeding tube	0.00	0.00	0.00	0.00	0.000	1.000
Weight loss	2.02	8.08	14.29	17.12	3.359	0.002
Weight gain	1.01	5.80	4.76	12.10	1.107	0.285

2D-CRT: two-dimensional conventional radiotherapy.

RT: radiotherapy.

CCRT: concurrent chemoradiotherapy.

SD: standard deviation.

EORTC QOL-C30: European Organization for Research and Treatment of Cancer

Quality of Life Questionnaire-Core 30.

EORTC QOL-H&N35: The EOTRC Quality of Life Questionnaire-Head and Neck 35.

**Table 4:** Mean values for all scales of RT versus CCRT with IMRT technique

Scales	RT (n = 22)		CCRT (n = 37)		T-test	*P*
	Mean	SD	Mean	SD		
EORTC QLQ-C30						
Global quality of life	86.31	12.49	79.04	16.42	1.481	0.146
Physical functioning	97.62	8.91	89.90	14.45	2.229	0.032
Role functioning	98.81	4.45	86.87	16.01	3.941	0.000
Emotional functioning	92.26	17.74	87.63	14.75	0.927	0.359
Cognitive functioning	95.24	10.19	84.34	13.14	2.764	0.008
Social functioning	98.81	4.45	87.88	14.60	3.895	0.000
Fatigue	4.76	7.18	15.82	20.04	−2.779	0.008
Nausea/emesis	2.38	6.05	0.51	2.90	1.107	0.285
Pain	5.95	8.29	9.09	13.24	−0.982	0.332
Dyspnea	2.38	8.91	6.06	19.46	−0.675	0.503
Insomnia	11.90	24.83	22.22	24.53	−1.314	0.196
Appetite loss	0.00	0.00	2.02	11.61	−0.647	0.521
Constipation	0.00	0.00	5.05	20.62	−0.911	0.367
Diarrhea	2.38	8.91	3.03	9.73	−0.214	0.831
Financial problems	7.14	14.19	27.27	25.62	−3.438	0.001
EORTC QLQ-H&N35						
Pain	2.38	5.09	3.79	5.55	−0.814	0.420
Swallowing	2.98	6.21	6.31	9.78	−1.404	0.169
Senses	8.33	8.65	13.13	16.01	−1.054	0.298
Speech	1.59	4.03	3.37	8.09	−0.779	0.440
Social contact	0.00	0.00	6.82	12.58	−3.114	0.004
Social eating	0.48	1.78	3.43	7.66	−2.089	0.043
Sexuality	4.76	10.19	27.78	20.27	−5.164	0.000
Teeth	7.14	14.19	16.16	20.62	−1.727	0.093
Opening mouth	2.38	8.91	9.09	15.08	−1.894	0.066
Dry mouth	19.05	21.54	24.24	26.71	−0.643	0.523
Sticky saliva	2.38	8.91	5.05	18.86	−0.504	0.617
Coughing	14.29	21.54	11.11	15.96	0.561	0.578
Feeling ill	2.38	8.91	11.11	19.84	−2.081	0.043
Pain killers	0.00	0.00	4.04	11.05	−2.101	0.044
Nutritional supplements	0.00	0.00	17.17	16.92	−5.831	0.000
Feeding tube	0.00	0.00	0.00	0.00	0.000	1.000
Weight loss	2.38	8.91	1.01	5.80	0.628	0.533
Weight gain	0.00	0.00	11.11	15.96	−4.000	0.000

IMRT: intensity-modulated radiotherapy.

RT: radiotherapy.

CCRT: concurrent chemoradiotherapy.

SD: standard deviation.

EORTC QOL-C30: European Organization for Research and Treatment of Cancer

Quality of Life Questionnaire-Core 30.

EORTC QOL-H&N35: The EOTRC Quality of Life Questionnaire-Head and Neck 35.

### Comparisons of QoL scales by different chemotherapy cycles

In the CCRT subgroup, 6 patients received 1 cycle of concurrent chemotherapy, 18 patients received 2 cycles of concurrent chemotherapy, and 27 patients received 3 cycles of concurrent chemotherapy. Survivors who received 1 cycle of concurrent chemotherapy had worse QoL outcomes than survivors who received 2 cycles of concurrent chemotherapy. Patients who received 3 cycles of concurrent chemotherapy had the best QoL outcomes (Table 5). Differences among most scales were clinically relevant. The unexpected results may indicate that survivors who are not tolerant of concurrent chemotherapy will have a worse QoL.

**Table 5:** Comparisons of mean values for all scales by different chemotherapy cycles

Scales	1 cycle CT (n = 6)		2 cycles CT (n = 18)		3 cycles CT (n = 27)		F-test	*P*
	Mean	SD	Mean	SD	Mean	SD		
EORTC QLQ-C30								
Global quality of life	54.17	10.21	66.67	13.71	72.12	18.85	3.023	0.058
Physical functioning	57.78	15.01	80.00	14.99	86.41	14.79	9.048	0.000
Role functioning	58.33	20.41	75.00	17.39	83.97	15.97	5.884	0.005
Emotional functioning	47.22	22.77	67.13	27.19	81.73	17.80	6.687	0.003
Cognitive functioning	36.11	16.39	69.44	22.32	78.21	13.96	13.836	0.000
Social functioning	47.22	16.39	65.74	23.20	85.26	15.15	12.769	0.000
Fatigue	50.00	15.32	33.95	24.84	19.66	21.15	5.493	0.007
Nausea/emesis	2.78	6.80	4.63	9.58	0.00	0.00	3.047	0.057
Pain	27.78	13.61	16.67	15.12	10.90	13.29	3.737	0.031
Dyspnea	16.67	18.26	5.56	12.78	8.97	22.23	0.785	0.462
Insomnia	55.56	17.21	40.74	24.40	24.36	29.15	4.309	0.019
Appetite loss	16.67	18.26	7.41	14.26	3.85	14.38	1.864	0.166
Constipation	0.00	0.00	1.85	7.86	7.69	23.68	0.798	0.456
Diarrhea	11.11	17.21	5.56	12.78	5.13	12.26	0.528	0.593
Financial problems	55.56	17.21	37.04	25.28	39.74	29.84	1.079	0.348
EORTC QLQ-H&N35								
Pain	13.89	6.80	7.87	6.69	7.05	8.06	2.070	0.138
Swallowing	36.11	13.61	20.37	14.64	10.26	10.62	11.381	0.000
Senses	30.56	19.48	19.44	16.42	12.82	16.54	2.927	0.063
Speech	14.81	9.07	4.32	9.44	5.13	9.55	2.998	0.059
Social contact	47.22	17.21	21.30	17.44	11.22	14.52	12.709	0.000
Social eating	21.11	14.25	4.07	7.97	4.87	8.55	8.724	0.001
Sexuality	91.67	20.41	48.15	28.52	28.21	17.49	20.327	0.000
Teeth	55.56	17.21	37.04	22.55	21.79	24.84	5.952	0.005
Opening mouth	44.44	17.21	20.37	16.72	14.10	23.42	5.291	0.008
Dry mouth	61.11	25.09	38.89	23.57	33.33	31.27	2.388	0.103
Sticky saliva	11.11	17.21	9.26	19.15	6.41	21.12	0.190	0.828
Coughing	27.78	13.61	11.11	16.17	11.54	16.17	2.811	0.070
Feeling ill	27.78	13.61	20.37	23.26	8.97	17.78	3.177	0.051
Pain killers	0.00	0.00	3.70	10.78	3.85	10.86	0.363	0.698
Nutritional supplements	22.22	17.21	18.52	17.04	19.23	16.79	0.109	0.897
Feeding tube	0.00	0.00	0.00	0.00	0.00	0.00	0.000	1.000
Weight loss	16.67	18.26	5.56	12.78	1.28	6.54	5.013	0.011
Weight gain	0.00	0.00	11.11	16.17	15.38	16.95	2.378	0.104

CT: chemotherapy.

SD: standard deviation.

EORTC QOL-C30: European Organization for Research and Treatment of Cancer Quality of Life Questionnaire-Core 30.

EORTC QOL-H&N35: The EOTRC Quality of Life Questionnaire-Head and Neck 35.

## DISCUSSION

The study suggests that RT has better outcomes in global QoL and functional scales of EORTC QLQ-C30 compared with CCRT. The result might help clinicians make better treatment decisions and provide information to health workers on which health services are most beneficial.

Different questionnaires were used for QoL assessment in NPC patients. A few studies used the EORTC QLQ-C30 questionnaire and the EORTC QLQ-H&N35 questionnaire [10–13]. Some studies used the Functional Assessment of Cancer Therapy-General (FACT-G) scale, the Functional Assessment of Cancer Therapy-Head and Neck (FACT-H&N) module [14, 15], and the Functional Assessment of Cancer Therapy-Nasopharyngeal (FACT-NP) subscale [16]. Other studies used the MOS 36-item short-form health survey (SF-36) [17, 18] and the University of Washington Quality of Life Questionnaire [19]. Recently, an NPC-specific scale (QoL-NPC) was developed to assess the physical functioning and health status of Chinese NPC patients [20]. However, FACT-NP has not been updated. SF-36 and the University of Washington Quality of Life Questionnaire are not specific questionnaires for QoL assessment in head-and-neck cancer patients, and QoL-NPC should be further evaluated by a large sample from different centers.

In this study, we used EORTC QLQ-C30 and the EORTC QLQ-H&N35 for QoL assessment because the two questionnaires are comprehensive. The EORTC QLQ-C30 contains a range of QoL issues related to different cancer patients, including head-and-neck cancer. The EORTC QLQ-C30 has been translated into many languages and is a widely used questionnaire. The QLQ-H&N35 is used to assess the QoL of patients with head-and-neck cancer specifically. The EORTC QLQ-C30 and the EORTC QLQ-H&N35 questionnaires are valid, internally consistency, and reliable in patients from different nations and were tested in large patient groups [21]. The Chinese version of the EORTC QLQ-C30 and the EORTC QLQ-H&N35 were previously tested, confirmed, and validated by some studies [11–13].

Our study showed no significant difference between RT and CCRT groups, except for weight gain reported in the EORTC QLQ-H&N35 questionnaire. The potential reasons are the following: (1) The EORTC QLQ-H&N35 might have some limitations in assessing QoL of NPC patients, although the EORTC QLQ-H&N35 is a specific questionnaire for assessing the QoL of head-and-neck cancer patients. NPC is different from other head-and-neck cancers because of its location, biological characteristics, and treatment. NPC survivors might experience deafness, otitis media, symptoms from temporal lobe injury, and hypopituitarism after radiotherapy. The EORTC QLQ-H&N35 does not deal with these adverse effects well enough. (2) CCRT was suggested to cause statistically significantly more acute toxic effects but similar late toxic effects compared with RT [4]. This outcome might be interpreted as the result of the few differences between RT and CCRT observed in the symptom scales of the EORTC QLQ-H&N35.

Previous studies mainly analyzed the effect of different radiotherapy techniques (IMRT vs. 2D-CRT) on QoL [11–13]. Only one study mentioned the effect of chemotherapy on QoL [10]. The above study found that concurrent chemotherapy adversely affected five symptom scales, but did not affect global QoL and functional scales. However, our study observed that concurrent chemotherapy adversely affected not only symptom scales but also global QoL and functional scales. Our results showed that CCRT had higher scores for fatigue and insomnia than did RT. Fatigue and insomnia might be caused by chemotherapy and contribute to loss of physical functioning, role functioning, and emotional functioning. The results of 2D-CRT and IMRT subgroup analysis further confirmed the above conclusion.

Some studies discussed the impact of financial problems on QoL [14, 22]. These studies found that financial difficulties adversely affect QoL. CCRT will increase the expenses of NPC treatment and eventually increase the financial difficulties of individuals in developing countries such as China. Consequently, CCRT adversely affects QoL. But, the relation between financial problems and QoL is still unclear. Further controlled studies should be performed to test the impact of financial difficulties on QoL.

Our result shows that CCRT adversely affects QoL. Thus, we hypothesize that patients who receive more cycles of chemotherapy will experience worse QoL. However, subgroup analyses of the effect of different chemotherapy cycles on QoL show an opposite result. Survivors who received 1 cycle of concurrent chemotherapy had worse QoL outcomes than survivors who received 2 cycles of concurrent chemotherapy. Patients who received 3 cycles of concurrent chemotherapy had the best QoL outcomes.

The potential interpretations are the following: (1) The 6 patients who received 1 cycle of concurrent chemotherapy received only 1 cycle because of serious toxicity during treatment. Serious toxicity made the 6 patients’ recovery worse. However, survivors who received 2 or 3 cycles of concurrent chemotherapy better tolerated chemotherapy and recovered better. (2) The 6 patients were all irradiated by 2D-CRT, the 18 patients who received 2 cycles of concurrent chemotherapy were treated with 2D-CRT or IMRT, and the 27 patients who received 3 cycles of concurrent chemotherapy were irradiated mostly by IMRT. Use of IMRT is associated with the reduction of physician-assessed late toxicities and improved patient-reported QoL in NPC survivors [11–13]. (3) Only 6 patients received 1 cycle of concurrent chemotherapy; thus the sample size of the CCRT group was insufficient. The result should therefore be treated with caution, and a large sample of patients should be investigated to verify the result.

The limitations of our study must be considered: (1) Only 106 patients were enrolled in our study, and the sample size of the CCRT group was insufficient for comparisons of QoL scales by different chemotherapy cycles. (2) The QoL measurement of our study was conducted at only one time point. A more methodologically sound approach is to use a longitudinal design in which the same individuals are assessed repeatedly at various time points.

## MATERIALS AND METHODS

### Study population

This cross-sectional study analyzed QoL data of patients with stage II NPC in the Cancer Hospital of Guangxi Medical University from June 2008 to June 2013. Inclusion criteria were (1) pathologically proved NPC, (2) stage II NPC per the 7th Edition of the UICC/AJCC staging system, (3) Karnofsky performance status >70, (4) receiving radical RT or CCRT, and (5) disease-free survival >3 years. Exclusion criteria were (1) age >70 or <18 years, (2) recurrent or metastatic NPC, (3) receiving induced or adjuvant chemotherapy, (4) a second malignancy, except for cured skin basal cell carcinoma or early stage cervical cancer, (5) severe cerebral, cardiac, hematologic, renal, hepatic, or mental disease, and (6) incompletion of the self-reporting questionnaire.

### Radiotherapy

Patients received 2D-CRT in two phases. In the first phase, patients were irradiated by 6-megavolt bilateral and opposing photon beams. The dose for faciocervical field and lower anterior cervical field was 36 Gy. In the second phase, the dose for primary tumor was boosted from 66 Gy to 70 Gy. The prescribed irradiation dose was 2 Gy per fraction with 5 daily fractions per week.

Patients received IMRT per the International Commission on Radiation Units and Measurements Report 62 guidelines. Gross tumor volume (GTVnx) and cervical lymph node tumor volume (GTVnd) were defined as gross shown by CT/MRI. Clinical target volume (CTV) included the GTV with a 1-cm to 1.5-cm margin, the entire nasopharyngeal space, and the positive lymph node regions. The prescribed radiation dose was 66 Gy to 70.06 Gy in 30 to 31 fractions for GTV, and 54 Gy to 60 Gy in 30 fractions for CTV with 5 daily fractions per week.

### Chemotherapy

Patients received concurrent chemotherapy on days 1, 22, and 43 during radiotherapy. Chemotherapy regimen was cisplatin 100 mg/m^2^/d by intravenous infusion. Chemotherapy was postponed or discontinued for patients who experienced serious toxicity and could not recover before the next schedule.

### QoL measurement

QoL assessment used the Chinese version of the EORTC QLQ-C30 questions and the EORTC QLQ-H&N35 questions [21, 23–25]. The EORTC QLQ-C30 is a cancer-specific questionnaire containing a global QoL score, five functional scales, three symptom scales, and six single items. The QLQ-H&N35 is a site-specific questionnaire assessing QoL of head-and-neck cancer patients. The QLQ-H&N35 contains seven multiple-item and six single-item scales. The standard score of all scales ranges from 0 to 100. A high score for a global QoL or functional scale represents a high/healthy level of global QoL or functioning, whereas a high score for a symptom scale represents a symptom problem. QoL changes of ≥10 points were considered clinically relevant [26, 27].

### Statistical analysis

Statistical analysis was performed using SPSS for Windows version 16.0 (SPSS Inc., Chicago, IL). The χ^2^ test was used for the comparisons of categorical data. The T-test was used to compare the mean scores of QoL between two groups. The F-test was used for the comparisons among groups. All significant tests were two-sided and *P* value <0.05 was considered statistically significant.

## CONCLUSIONS

This study suggests that CCRT degrades broad aspects of QoL for patients with stage II NPC. RT may be a better treatment choice for stage II NPC compared with CCRT. However, undetected factors still might be related to QoL. The data in this study must be tested, preferably in a prospective, randomized trial.
